# Lipid Anchoring Improves Lubrication and Wear Resistance of the Collagen I Matrix

**DOI:** 10.1021/acs.langmuir.1c01581

**Published:** 2021-11-17

**Authors:** Hui Yuan, Hsiu-Wei Cheng, Laura LE Mears, Renliang Huang, Rongxin Su, Wei Qi, Zhimin He, Markus Valtiner

**Affiliations:** † State Key Laboratory of Chemical Engineering, Tianjin Key Laboratory of Membrane Science and Desalination Technology, School of Chemical Engineering and Technology, Tianjin University and Collaborative Innovation Center of Chemical Science and Engineering (Tianjin), Tianjin 300072, China; ‡ Institute of Applied Physics, Vienna University of Technology, Vienna 1040, Austria; § School of Marine Science and Technology, Tianjin University, Tianjin 300072, China

## Abstract

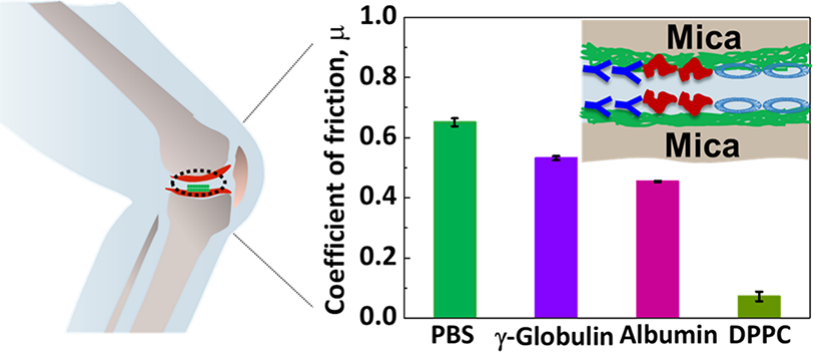

Osteoarthritis is a prevalent degenerative joint disease characterized by progressive articular cartilage loss and destruction. The resultant increase in friction causes severe pain. The collagen I matrix (COL I) has been used clinically for cartilage repair; however, how COL I acts at cartilage surfaces is unclear. Here, we studied adsorption and lubrication of synovial fluid components, albumin, γ-globulin, and the phospholipid DPPC, on COL I under physiological conditions using surface plasmon resonance and an in situ sensing surface force apparatus. Our results revealed COL I had poor lubrication ability, a fairly high coefficient of friction (COF, μ = 0.651 ± 0.013), and surface damage under a 7 mN load. DPPC formed an improved lubricating layer on COL I (μ = 0.072 ± 0.016). In sharp contrast, albumin and γ-globulin exhibited poor lubrication with an order of magnitude higher COF but still provided benefits by protecting COL I from wear. Hence, DPPC on COL I may help optimize COL I implantation design.

## Introduction

Osteoarthritis (OA) is the most common degenerative and highly prevalent joint disease,^1−4^ which is characterized by progressive loss and destruction of the articular cartilage extracellular matrix.^1−8^ The treatment of osteoarthritis is a major focus in medicine, particularly to regenerate damaged articular cartilage, since the self-repair capability of damaged cartilage tissue is very limited: cartilage has an avascular structure and low cell content.^9^ Nowadays, the collagen I matrix (COL I) is widely used as a supportive framework for cartilage repair in the clinic,^10−14^ where the physiological compatibility of recovered cartilage tissue is similar to that of the healthy hyaline cartilage tissue.^11^

Although the COL I provides a good support to regenerate the cartilage tissue, immobilizing the material on the damaged site after the implantation is a big challenge. The friction from daily activities or recovery exercises leads to displacement, wear, or detachment.^10−14^ Significantly limiting joint activity helps with stabilizing the implant at an early stage; however, this will prolong the duration of disability for the patient that may further cause muscle wastage. Therefore, how to design a mechanically stable and low friction treatment that resolves the issues addressed above inspired us to study the lubrication properties of COL I in a model articular cartilage system.

Articular cartilage is a highly efficient water-based lubrication system with a sliding coefficient of friction (COF) of 5 × 10^–4^ and can support up to 20 MPa of normal pressure.^15−18^ The efficiency of such a lubrication system is strongly influenced by the synovial fluid (SF) that mediates the shear plane properties with different surface-adsorbed lubrication molecules, i.e., surfaces slide past each other along the plane of adsorbed molecules rather than along a direct contact.^19^

Natural biomolecules such as albumin,^20−22^ γ-globulin,^20−23^ and 1,2-dipalmitoyl-*sn*-glycero-3-phosphatidylcholine (DPPC)^24^ are known principal components in physiological SF (shown in Table 1) and have been demonstrated to be good boundary lubricants. Among the mentioned components, albumin and γ-globulin have been reported to have good lubrication capability on the surface of ceramic/polyethylene joint implants by Ghosh et al.^25^ Similarly, DPPC has also been reported as a good lubricant on mica under a high pressure of 566 atm between two mica surfaces and on a hyaluronan surface with anchored DPPC.^26−28^ Although the lubrication properties of albumin, γ-globulin, and DPPC have been well characterized on mica and other mentioned substrates, their adsorption and boundary lubrication on COL I surfaces have not yet been clarified.

**Table 1:** Lubricants’ Concentration and the First-Order Adsorption Rate Constant (*k*) on COL I Surfaces in Normal Physiological Synovial Fluid (pH = 7.4)^33−35^

lubricant	concentration (mg/mL)^20−24^	*k* (s^–1^)	*R*^2^
albumin	∼11	0.262	0.96
γ-globulin	∼7	0.100	0.98
DPPC	∼0.1	0.005	0.95

**Scheme 1:**
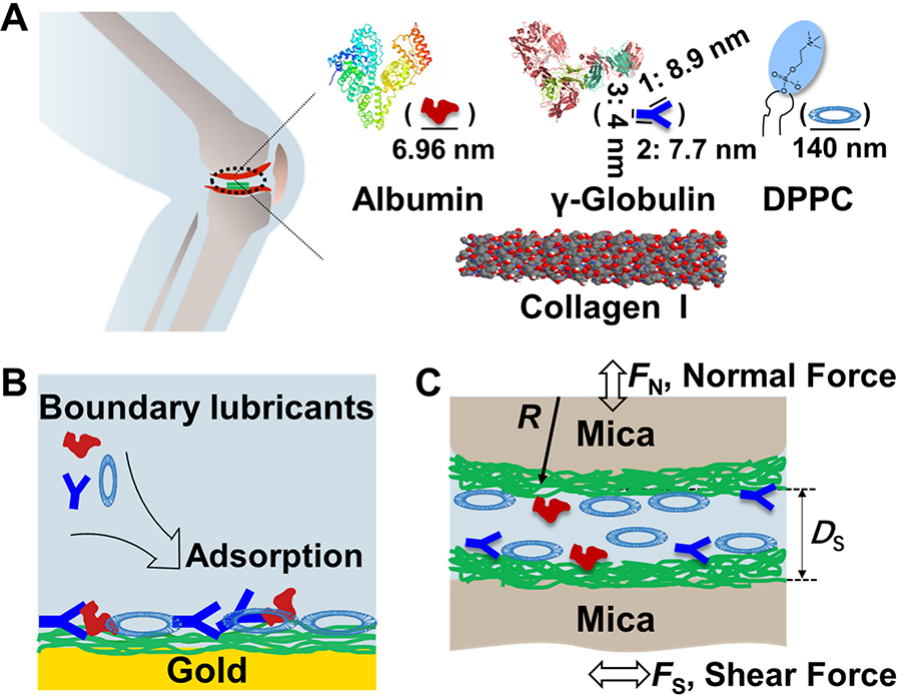
(A) Schematic Illustration of the Cartilage Surface and the Structures of Albumin (the Stokes Radius of Albumin is 3.48 nm),^29^ γ-Globulin (the Lengths of Regions 1, 2, and 3 are 8.9, 7.7, and 4 nm, Respectively),^30^ DPPC, and collagen I; Schematic Illustration of Albumin, γ-Globulin, and DPPC on (B) COL I-Coated Gold or (C) Mica Model Surfaces, Mimicking the Major Protein and Lipid Components in a Synovial Fluid System Adsorbed onto the Collagen Matrix,^20^ i.e., the Collagen I Matrix was Added First Followed by the Components That Might Adsorb onto the Matrix The structure of albumin, γ-globulin, and collagen I were obtained from the Protein Data Bank.^31^

In this study, we investigated the adsorption behavior of boundary lubricants (illustrated in Scheme 1), including albumin, γ-globulin, and DPPC, in a physiological synovial fluid mimic on COL I. Further, to the adsorption characteristics, we studied how the adsorbed albumin, γ-globulin, and DPPC affects the lubrication behavior between two collagen I surfaces.

Complementary, surface plasmon resonance spectroscopy (SPR) was employed to monitor the adsorption–desorption process of boundary lubricants in real time. The equilibrium adsorption (*q_e_*), irreversible adsorption (*q_ir_*), and the adsorption kinetics under different conditions were calculated for the boundary lubricants on a collagen I surface. The surface force apparatus (SFA) was used to measure the interactions, including normal and shear forces, between collagen I and synovial fluid-adsorbed boundary lubricants.

## Materials and Methods

### Materials

Collagen type I (stock concentration of 5.82 mg/mL in 0.1 M acetic acid) was isolated from muscle tendon of mice by Arthro-Anda Tianjin Biologic Technology Co., Ltd. (Tianjin, China). Bovine serum albumin (BSA) (closely related to human serum albumin in structure, size, and composition) and γ-globulin were obtained from Aladdin (Shanghai, China). 1,2-Dipalmitoyl-*sn*-glycero-3 phosphatidylcholine (DPPC, 16:0) was purchased from Sigma-Aldrich (Tianjin, China) with >99% purity. The PBS buffer was prepared by the formula (137 mM NaCl, 10 mM phosphate salt, and 2.7 mM KCl) at pH 7.4 confirmed using a pH meter and stored at 4 °C before use. The water used in all the experiments was purified using a three-stage Millipore Milli-Q Plus 185 purification system (Millipore Corp., Bedford, MA). The pH values of all the solutions used were determined using an MP220 pH meter (Mettler-Toledo, Switzerland). All the solutions were filtered using syringe filters with 0.22 μm-diameter pores.

### Preparation of Synovial Fluid Components

To mimic physiological concentrations in synovial fluid, bovine serum albumin, γ-globulin, and DPPC were dissolved in phosphate-buffered saline (PBS, 10 mM) solution (pH 7.4) to concentrations of 11, 7, and 0.1 mg/mL, as shown in Table 1.

### Preparation of Substrates for Adsorption/Desorption Measurements

Before the experiment, the gold (Au) chips were washed with ethyl alcohol and ultrapure water (Milli-Q) sequentially, dried by nitrogen gas, then cleaned with UV/ozone treatment for 15 min, and put in a freshly prepared solution of a 5:1:1 mixture of ultrapure water, ammonia solution (NH_3_), and hydrogen peroxide (H_2_O_2_) at 70 °C for 10 min. Finally, the chips were rinsed with ethyl alcohol and ultrapure water (Milli-Q) sequentially, dried by nitrogen gas, and treated with a UV/ozone treatment for 2 h. All the measurements were conducted at 25 °C.

The preparation of the collagen type I fibril suspension was as follows: 5.82 mg/mL collagen type I in 0.1 M acetic acid was diluted 120 times with phosphate-buffered saline (PBS, 10 mM) solution, giving a concentration of 48 μg/mL, which was used clinically for articular cartilage repair. The collagen type I film was formed by the following steps: (i) 10 mM PBS buffer solution was flowed across the chips’ surfaces at a rate of 30 μL/min for approximately 40 min to create a baseline for the measurements; (ii) the collagen type I fibril suspension dissolved in 10 mM PBS buffer solution was flowed over the chip at a rate of 5 μL/min to adsorb, until reaching a plateau for 36 min; (iii) the chip was rinsed by 10 mM PBS buffer solution to remove unattached molecules and obtained the collagen type I film layer in a physiological state for further experimentation.

### Preparation of Collagen I Layers onto Mica Surfaces

To obtain the collagen I-coated mica surface, first, two freshly cleaved atomically smooth back-silvered mica sheets were glued onto cylindrical disks (made of quartz, *R* = 1–2 cm) by UV curing glue, NOA81 (Norland adhesives, UV-curable adhesive), and kept in the UV light for about 3 h, and then 100 μL of COL I solution at 48 μg/mL was dropped onto the mica surfaces and incubated for 1 h and then rinsed with PBS to block nonspecific interactions to form the collagen I layer. Mica–mica contact in air was recorded on a spectrometer to set the separation distance *D* = 0 prior to each surface functionalization.

### Adsorption/Desorption Measurements by SPR Measurement

To assess the interaction between this collagen I matrix and the different synovial fluid components, a biacore X100 was used to measure the real-time nonspecific adsorption of the different synovial fluid components on the surface of the collagen I matrix mimicking the articular cartilage repair. After the COL I surface modification, then (i) different synovial fluid components were flowed over the collagen type I film layer at a rate of 5 μL/min to adsorb by injecting twice for about 36 min; at this step, the following measurements for different synovial fluid components were carried out, including (1) the adsorption behavior of protein on COL I surfaces (albumin alone or γ-globulin alone) and (2) the adsorption behavior of lipid on COL I surfaces (DPPC alone); (ii) finally, the chip was rinsed by 10 mM PBS buffer solution to remove unattached synovial fluid molecules.

### Surface Force and Lubrication Measurement

The surface force apparatus (SFA) is a scientific instrument, which measures the interaction force between two surfaces with a separation distance and normal force accuracy of 1 Å and 10 nN, respectively. Mica sheets in this study were hand cleaved, and the edges of the sheets were melt-cut with a hot platinum wire. The obtained mica sheets were back-silvered with nominal 40 nm thickness. The SFA used at TU Wien was home-built with two perpendicular load cells (stiff springs with strain gauges) to measure the forces directly simultaneously with the distance measurement using white light interferometry.^32^ The interference pattern, fringes of equal chromatic order (FECO), is captured on a 2D CCD sensor depicting a cross section of the contact area. The top surface can be moved both in the normal and shear directions using two piezoes.

For the normal and friction force measurements, after forming the modified COL I surface, different synovial fluid components including PBS as a control, albumin, γ-globulin, or DPPC were flowed into the cell to adsorb for about 36 min; then, we measured normal and shear forces. For the normal force, we used a 3–6 nm/s approach speed during a force run. For friction force measurements, the first step is adjusting the alignment to ensure that the surfaces remain parallel during shear to ensure experiment reproducibility. The surfaces are mounted on a goniometer, and it is adjusted to hold the FECO steady during sliding motion over 10 μm at a large distance. We adjust the two surfaces to within a 10 nm tilt over a 10 μm motion, which is a misalignment angle of a maximum of 1.7 × 10^–5^ degrees.^32^ Shear force was generated via the shear piezo, and the shear velocity was 0.3 μm/s. Film pressures were obtained from FECO by , *F*_⊥_ is measured directly by recording the strain gauges’ data, and the radius of the contacted area can be obtained from the FECO shape, and then one can calculate the pressure directly.

All normal force and friction force measurements were performed at 25 °C and repeated in two to three independent experiments at 3–5 positions. For every force run, it returns to the same hard wall position within error, indicating that there is no material loss or exchange between the surfaces. The experimental results at different contacting positions are shown in Figure S1. The SFA data analysis was performed with SFA Explorer.^32^

## Results and Discussion

### Preparation of the Collagen I Matrix Surface

**Figure 1:**
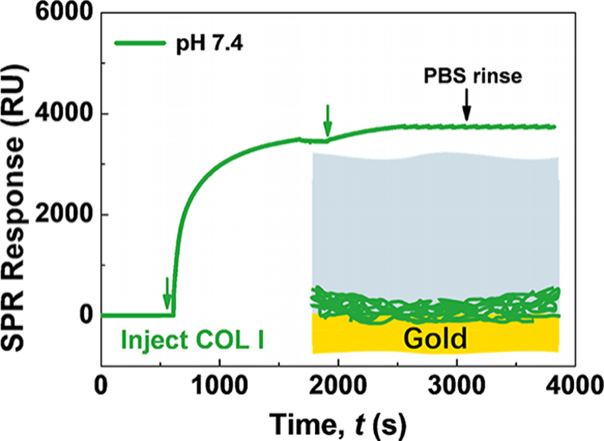
SPR sensorgram showing the real-time adsorption of COL I at 48 μg/mL (the concentration used clinically) on Au chips in physiological pH 7.4.

To obtain a collagen I matrix surface, an aqueous solution of collagen I was flowed over the gold (Au) chip surface with the SPR equipment. As shown in Figure 1, the SPR signal response remained constant after a second injection of material, indicating that the Au surface was fully covered by COL I. During the final rinsing with PBS, there was no decrease in the SPR signal, suggesting that COL I was strongly bound to the Au surface with irreversible adsorption and no COL I was washed off. The adsorption amount of COL I at physiological pH 7.4 on Au surfaces was approximately 340 ng/cm^2^, and the obtained COL I film thickness was about 3.24 nm (seen in Table S1).

### SPR Measurement

**Figure 2:**
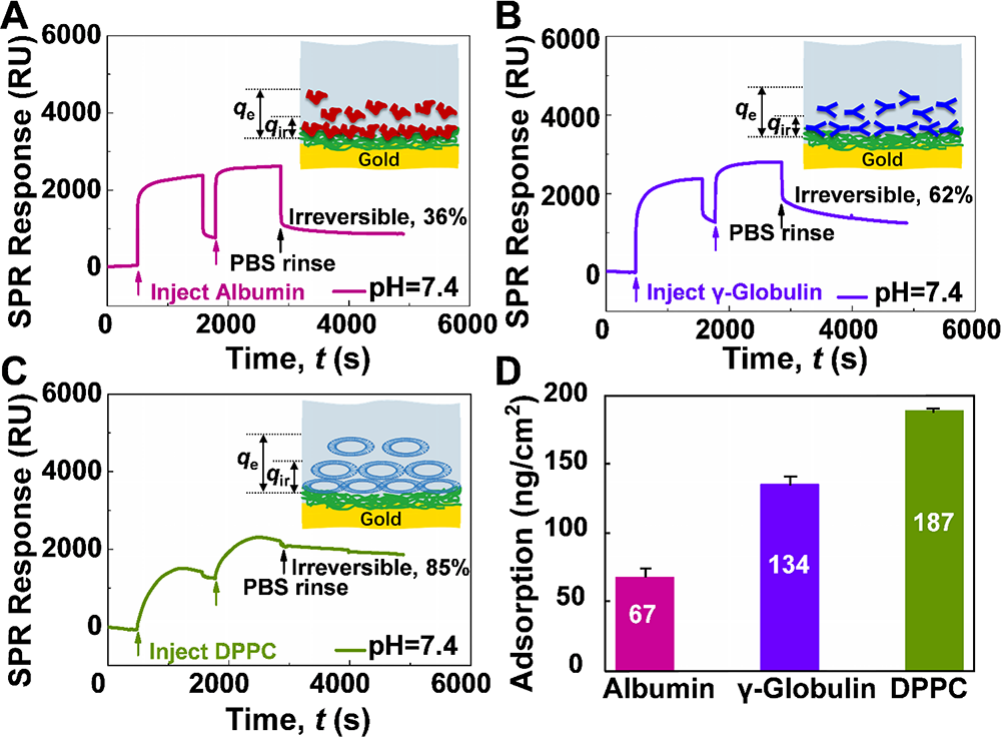
SPR sensorgrams showing the real-time change in mass of (A) albumin, (B) γ-globulin, and (C) DPPC and (D) adsorbed amount (*q_ir_*) of albumin, γ-globulin, and DPPC on the COL surface with two injections of the physiological synovial fluid (pH 7.4) mimic.

Figure 2A–D shows the SPR sensorgrams of the real-time change in adsorption amount of albumin, γ-globulin, and DPPC on these COL I model surfaces with two injections of physiological synovial fluid (pH 7.4) mimic. As shown in Figure 2A, upon injection of albumin, the SPR signal increased rapidly, indicative of the rapid adsorption of albumin on the COL I surface. After two injections, the *q_e_* value reached 186 ng/cm^2^. Further washing with PBS solution led to a remarkable decrease in the SPR signal, achieving a *q_ir_* value of 67 ng/cm^2^ (seen in Figure 2D), accounting for 36% of the *q_e_* value.

Similar SPR sensorgrams were also found in the case of γ-globulin (Figure 2B), which had a similar *q_e_* value of 216 ng/cm^2^ but a higher *q_ir_* value of 134 ng/cm^2^ (seen in Figure 2D) (∼62% of the *q_e_* value) than albumin. For DPPC, as shown in Figure 2C, the *q_e_* value also reached 220 ng/cm^2^ after two injections. The SPR signal decreased slightly during washing with PBS solution. The *q_ir_* value was 187 ng/cm^2^ (seen in Figure 2D), accounting for 85% of the *q_e_* value, indicating that DPPC molecules were mainly bound to the COL I surface via irreversible adsorption.

According to the SPR sensorgrams, we calculated the first-order adsorption rate constant (*k*) of boundary lubricants on COL I surfaces by data fitting with the Lagergren equation (seen in Table S2).^23,24^ As summarized in Table 1, the albumin had a *k* value of 0.262 s^–1^ at pH 7.4, which is much higher than γ-globulin (0.1 s^–1^), while the DPPC had a very low *k* value of 0.005 s^–1^, indicating that the adsorption of DPPC is very slow.

As the next step, to investigate whether the adsorbed SF boundary lubricants improve the lubrication and contribute to the wear protection of COL I, we used an sSFA to measure the normal and shear forces between collagen I-modified surfaces (on mica) in PBS as a control and COL I additionally treated with albumin, γ-globulin, or DPPC as SF mimics at physiological pH = 7.4 at 25 °C.

First, we performed normal and friction measurements on the collagen I matrix in PBS. Briefly, the experiments were performed as follows: We established the FECO position for the mica–mica distance *D* = 0 in air followed by the dry contact of collagen I-coated surfaces. From the FECO fitting, the thickness of the collagen I layer on mica gave 2.67 ± 0.2 nm, consistent with the thickness obtained from the SPR measurement, as shown in Table S1. Then, we injected PBS into the cell.

**Figure 3:**
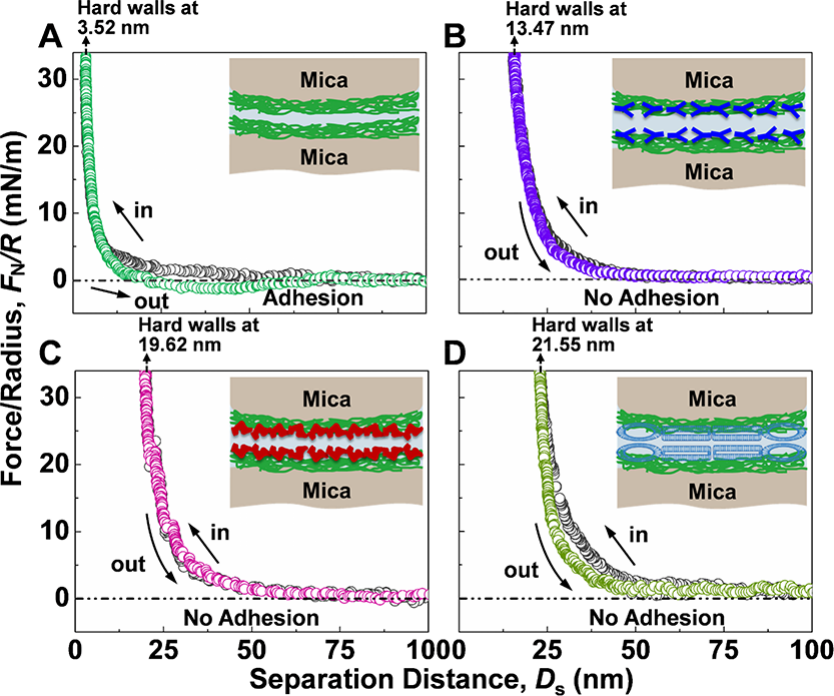
Normal force *F*_N_ normalized by the radius of curvature *R* between collagen I-coated surfaces (A) in PBS, (B) with γ-globulin, (C) with albumin, and (D) with DPPC as a function of the film thickness *D*.

**Figure 4:**
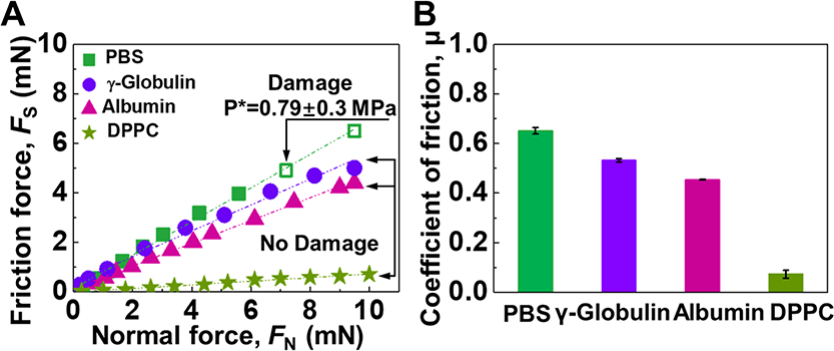
(A) Friction force *F*_s_ as a function of normal force *F*_N_ between (i) collagen I-coated surfaces in PBS only (square), (ii) with γ-globulin (circle), (iii) with albumin (triangle), and (iv) with DPPC (pentagram). The surfaces were all sheared at sliding velocities of ν = 0.3 μm/s and repeated three to five times with different contact positions. Open symbols indicate measurements after the surfaces became damaged. (B) The coefficient of friction (COF) for each of the additives and none (PBS) on the collagen I adsorbed on mica.

As shown in Figure 3A and Figure S1A,A′, the measured normal forces were weakly adhesive, with a long-range but weak adhesive plateau that disappeared at about 75 nm. We interpret this long-range attractive interaction as entangling of the molecularly overlapping COL I layers from both sides. The friction force *F*_s_ was measured as a function of the normal force and expectedly showed very poor lubrication properties. As shown in Figure 4, the friction force rapidly increased with the increasing load, exhibiting a fairly high coefficient of friction (μ_1_ = 0.651 ± 0.013). This high friction force could be due to the adhesive force, and hence molecular intertwining, between collagen I layers in the process of shearing. The two collagen I layers can be conglutinated repeatedly during shear and may remain in the conglutination state with the increasing loading, giving rise to the friction force and resulting damage. Specifically, collagen I surface damage was observed at a loading of about 7 mN (at a pressure of ∼0.79 MPa).

To quantify the lubrication behavior of adsorbed γ-globulin on collagen I layers, we injected 7 mg/mL γ-globulin into the solution with the collagen I surfaces well separated. After allowing adsorption for 36 min, the two surfaces were brought together without changing the contact position. By comparing the hard wall positions (the contact between the two opposing mica surfaces is set to *D* = 0, a hard wall means the distance that two surfaces can approach to) of the collagen I layers before and after injecting the γ-globulin, see again Figure 3A,B (also Figure S1B,B′), the thickness of the adsorbed γ-globulin was estimated to be about 8.78 ± 0.27 nm. The measured absolute thickness allows us to derive the surface adsorption geometry of γ-globulin. As shown in Scheme 1, we know that γ-globulin is composed of regions 1, 2, and 3 arranged in the typical “Y” shape. Because the γ-globulin is adsorbed to both collagen I surfaces, the only symmetric orientation that can explain the thickness of 9.95 nm is a single layer on each COL I surface, lying flat on the surface, with an approximate thickness of region 3 (4 nm).^30,36^

As shown in Figure 3B and Figure S1B,B′, after adsorption of γ-globulin, the normal interaction force changed from adhesive to repulsive. Also, the friction forces *F*_s_ monitored on collagen I layers in the γ-globulin solution were found to exhibit a moderately lower coefficient of friction (μ_2_ = 0.532 ± 0.007) shown in Figure 4. Surface damage was not detected in experiments, which indicates, in addition to the slightly reduced COF, that most importantly, the adsorbed γ-globulin offers protection of the collagen surfaces against wear. It is plausible that the adsorption of γ-globulin prevents intertwining of the opposing COL I matrix layers, which in turn also prevents damage formation. Compared to a previous work on ceramic surfaces, γ-globulin is, however, not a good lubricant.^25^

We next turn to the adsorbed albumin; similarly, we injected 11 mg/mL albumin into the cell and allowed adsorption to the COL I surfaces for 36 min. From Figure 3C and Figure S1C,C′, we note that the adsorbed albumin changed the adhesive force of collagen I layers to a repulsive force out to a separation of about 80 nm. The hard wall was about 19.68 ± 0.1 nm, indicating that the thickness of adsorbed albumin was about 14.34 nm. Hence, the adsorbed albumin was one layer thick on each collagen I surface (the Stokes radius of albumin is 3.48 nm).^29,37−40^ The friction forces *F*_s_ monitored on collagen I in albumin were found to exhibit a further lowering of the coefficient of friction (μ_3_ = 0.454 ± 0.002) shown in Figure 4. It is also worth noting that no surface damage was detected. The adsorbed albumin can also both decrease the coefficient of friction and protect the collagen I from damage, likely by a similar mechanism discussed for γ-globulin. In addition, the longer-ranged repulsive forces may indicate a slight swelling of the hydrated COL I matrix. Yet again, the data indicates no excellent lubrication properties of albumin in comparison to albumin effects found for ceramic surfaces.

In a third set of experiments, instead of albumin, we injected 0.1 mg/mL DPPC between the well-separated collagen I surfaces. After adsorption for 36 min, the two surfaces were brought together. As shown in Figure 3D and Figure S1D,D′, the injection of DPPC solution changed the interaction of the COL I surfaces from adhesive to a repulsive force out to a 50 nm separation. At the highest pressures accessed in our study, the hard wall was at 22.39 ± 0.56 nm, which means that the thickness of COL I with adsorbed DPPC was about 17.05 nm, corresponding to approximately four bilayers since the thickness of a single bilayer is about 3–5 nm.^28^ Consistent with the longer-ranged repulsive force during the approach, this may indicate the adsorption of DPPC liposome layers that can be compressed into a flat bilayer shape at the highest loads. Also, AFM imaging shown in Figure S2 indicates the adsorption of intact vesicles on the surfaces. The friction forces *F*_s_ on collagen I layers in DPPC indicate a significant change of the lubrication properties, which was found to exhibit the lowest coefficient of friction (μ_4_ = 0.072 ± 0.016), as shown in Figure 4. There was no surface damage detected, which indicates that adsorbed DPPC has an improved lubrication ability and protects the collagen I.

## Conclusions

To conclude, using surface plasmon resonance spectroscopy, we first studied the adsorption behavior of synovial fluid boundary lubricants, namely, albumin, γ-globulin, and DPPC, on a collagen I matrix in a physiological synovial fluid mimic. Our primary findings are the following: albumin, γ-globulin, and DPPC can form an adsorbed boundary layer on COL I, and the order of adsorbed masses was DPPC > γ-globulin > albumin, although the rate of adsorption was reversed: albumin > γ-globulin > DPPC.

Using the SFA, we studied the lubrication behavior of the same adsorbed boundary lubricants under the same conditions. Our main findings are (i) the collagen I matrix alone has poor lubrication properties with a high coefficient of friction (μ = 0.651 ± 0.013) and surface damage occurred at a loading of 7 mN; (ii) all adsorbed SF components, that is, albumin, γ-globulin, and DPPC, can reduce the coefficient of friction and protect the collagen I matrix against wear. Their ability to improve the lubrication was in the order DPPC > albumin > γ-globulin; and (iii) more specifically, DPPC has a very good lubrication ability, with a coefficient of friction of μ = 0.072 ± 0.016. In sharp contrast, both albumin and γ-globulin exhibit poor lubrication characteristics with a COF an order of magnitude higher but still provide the beneficial property of protecting COL I from wear, presumably due to blocking the surface, and preventing intertwining of apposing COL I layers.

The friction coefficient is the key to determining the tribological properties, and in clinical applications, a low friction coefficient is desirable for a long and effective repair using joint implantation matrices. Effective boundary lubrication depends on the adsorbed surface layers adhered to the cartilage surface. From our results, anchoring a DPPC layer on a COL I surface after its implantation may potentially be an efficient approach to reduce the wear of cartilage and improve the therapeutic effect of COL I.

Our adsorption and lubrication measurements provide a good understanding of the interaction mechanism between SF boundary lubricants with COL I in an articular cartilage mimic system and will help improve collagen I matrix materials. Significantly, here we also put forward an efficient way to anchor a DPPC layer on the COL I surface after its implantation to improve its therapeutic effect.

## Supporting Information Available

DPPC liposomes’ preparation, characterization and analysis of atomic force microscopy and contact angle, and calculated first-order adsorption rate constant (*k*) (PDF)
