# Supplementary material for: Lipid Anchoring Improves Lubrication and Wear Resistance of the Collagen I Matrix
Source: Langmuir. 2021 Nov 17;37(47):13810–5. doi: 10.1021/acs.langmuir.1c01581 (PMC8638261; doi:10.1021/acs.langmuir.1c01581)
Supplement: Supplementary file 1 — la1c01581_si_001.pdf [file la1c01581_si_001.pdf]

Supporting Information

Lipid anchoring improves lubrication and wear resistance of collagen I matrix

Hui Yuan[†,‡], Hsiu-Wei Cheng[‡], Laura LE Mears[‡], Renliang Huang[§], Rongxin Su[†,], Wei Qi[†], Zhimin He[†], Markus Valtiner[‡,]**

[†] State Key Laboratory of Chemical Engineering, Tianjin Key Laboratory of Membrane Science and Desalination Technology, School of Chemical Engineering and Technology, Tianjin University and Collaborative Innovation Center of Chemical Science and Engineering (Tianjin), Tianjin 300072, China

[‡] Institute of Applied Physics, Vienna University of Technology, Vienna 1040, Austria

[§] School of Marine Science and Technology, Tianjin University, Tianjin 300072, China

---

* Author to whom any correspondence should be addressed

E-mail: surx@tju.edu.cn (R.S.); markus.valtiner@tuwien.ac.at (M.V.)

Tel: +86 22 27407799. Fax: +86 22 27407599.

Tel: +43 1 58801 13440. Fax: +43 1 58801 13499.

■ MATERIALS AND METHODS

The DPPC liposomes preparation. Multilamellar vesicles (MLVs) were prepared by extrusion using a mini-extruder (Avanti Polar Lipids Inc, USA). Briefly, DPPC was dissolved with PBS, and hydrated about 15 min at 70-75°C (well above its solid-ordered to liquid disordered transition temperature TM(DPPC) 41°C). Then, MLVs were downsized to form single-unilamellar vesicles (SUVs) by stepwise extrusion through polycarbonate membranes starting with a 400 nm (4 times) 100 nm (4 times) and ending with 50nm-pore-size membrane (7 times)[1-2].

Atomic force microscopy (AFM) characterization. The image of prepared DPPC solution at a concentration of 0.1 mg/mL in PBS (10 mM, pH 7.4) on mica surface was carried out with a Nano surf C3000 Controller instrument (Asylum Research, Santa Barbara, CA) at 25℃. Scanning in tapping mode used a Tap 190Al-G tip (Budget Sensors) with a nominal spring constant of 48 N/m.

Contact angle (CA) characterization. The static contact angle of collagen type I at a concentration of 48 μg/mL in pH 7.4 (PBS, 10 mM) on Au was measured using a JCY contact angle measuring device (Shanghai Fangrui Instrument Co., Ltd, China) and was calculated by fitting the profile of the observed equilibrium CA of a droplet from the three-phase interface of the image captured using a CCD camera. The volume of the water droplet was 1 μL for each test and the average CA values were obtained from measurements taken at four different positions on the same substrate at ambient temperature.

■ RESULTS AND DISCUSSION

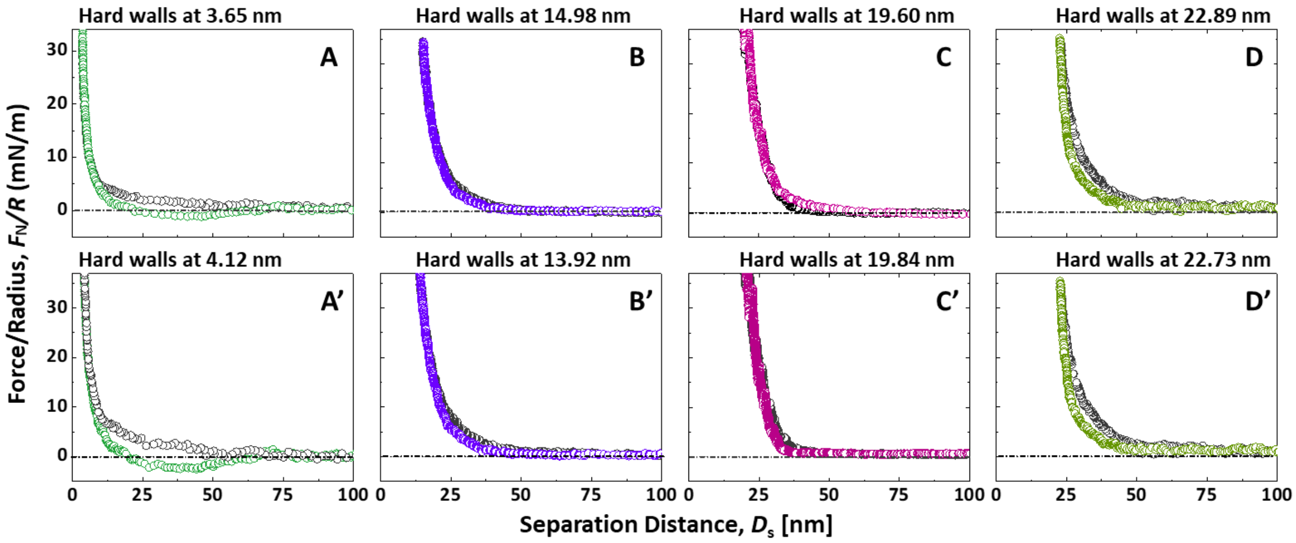

Fig S1 The experiment results of normal force F_N normalized by the radius of curvature R at different contacting positions between collagen I coated surfaces (A and A') in PBS, (B and B') with γ-globulin, (C and C') with albumin, and (D and D') with DPPC, as a function of the film thickness Ds.

AFM analysis of DPPC liposome on a mica surface. AFM images of a mica surface coated with an adsorbed layer of DPPC liposomes were measured in PBS solution using a Tap 190Al-G tip with a nominal spring constant of 48 N/m. From Fig S2, we can see we obtain the liposomes successfully using the extruder.

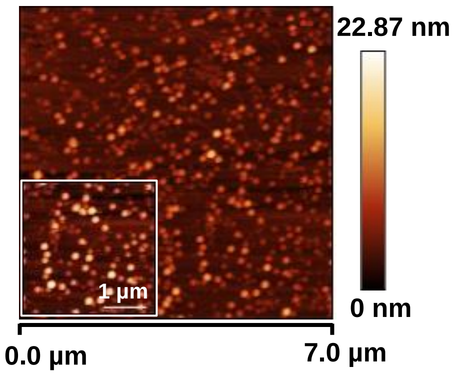

Fig S2 AFM images of mica surfaces coated with adsorbed layers of DPPC liposomes which were measured in PBS solution using a Tap 190Al-G tip with a nominal spring constant of 48 N/m, insert

shows a different contrast image of the surface. Scale bar in insert corresponds to 1 μm.

Contact angle analysis of COL I film on a gold surface. To characterize the wettability of the COL I film-coated gold surface, the contact angles of Au-COL I surface was measured. As the inset in Table S1 shows, the COL I-coated gold surface had a water contact angle of 24.91°. The typical contact angle of gold is 95.4 degrees, which further confirms the modification by COL I.

Table S1 The thickness and contact angle of modified COL I film on gold surface in physiological pH 7.4.

	**Film thickness**	**Contact angle**	
COL I	3.24±0.2 nm	24.91±0.3°	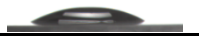
Au	n/a	95.4±1.2°	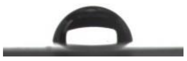

The first order adsorption rate constant (*k*) of boundary lubricants on COL I surface. According to the SPR sensorgrams, we calculated the first order adsorption rate constant (k) of boundary lubricants on COL I surface by data fitting with Lagergren equation, seen in Table S2. As shown in Fig 1 (modified amount) and table S1 (thickness, contact angle), we obtained a stable COL I coated surface in physiological pH 7.4. Hence, the adsorbed rate of synovial fluid boundary lubricants on COL I surface was not affected by COL I. In this article, the Lagergren quasi first order rate equation was used to fit the experimental data to explain the adsorption mechanism. The Lagergren is following:

$$\frac{dx}{dt} = k_1(X - x) \qquad (1)$$

where x is the adsorption amount (ng/cm^2) at time, t (s), X is the adsorption amount (ng/cm^2) at

equilibrium,

Integrate the (1) expression, when t=0, x=0, t=t, x=x,

$$\int_{x=0}^{x=x} \frac{1}{X-x} dx = \int_{t=0}^{t=t} k_1 dt \tag{2}$$

$$\ln \frac{X}{X-x} = k_1 t \tag{3}$$

According to the symbol adopted in this paper, the expression of the pseudo-first-order adsorption rate equation was:

$$\ln \left(\frac{q_e}{q_e - q_t} \right) = k_1 t \tag{4}$$

where q_t was the adsorption amount at t, q_e was the adsorption amount at equilibrium, k_1 was first order adsorption rate constant, s^{-1}

Table S2 The adsorbed rate equation of boundary lubricants on COL I surface was fitted using the Lagergren equation as described below in physiological pH 7.4.

**Lubricants**	**Equation**
Albumin	$\ln \frac{5483.92}{5483.92 - q_t} = k_1 t$
γ-Globulin	$\ln \frac{5359.38}{5359.38 - q_t} = k_1 t$
DPPC	$\ln \frac{2921.26}{2921.26 - q_t} = k_1 t$

■ REFERENCES

1. Goldberg, R.; Schroeder, A.; Silbert, G.; Turjeman, K.; Barenholz, Y.; Klein, J., Boundary lubricants with exceptionally low friction coefficients based on 2D close-packed phosphatidylcholine liposomes. *Adv. Mat.* **2011,** *23* (31), 3517-3521.

2. Zhu, L. Y.; Seror, J.; Day, A. J.; Kampf, N.; Klein, J., Ultra-low friction between boundary layers of hyaluronan-phosphatidylcholine complexes. *Acta Biomater.* **2017,** *59*, 283-292.
